# Efficacy of Botulinum Toxin for Treating Sialorrhea in Neuromuscular Conditions

**DOI:** 10.3389/fneur.2020.00513

**Published:** 2020-06-10

**Authors:** Harsh Singh, Yash Nene, Tejas R. Mehta, Raghav Govindarajan

**Affiliations:** ^1^Department of Neurology, University of Missouri, Columbia, MO, United States; ^2^Assistant Professor of Neurology, Department of Neurology, University of Missouri, Columbia, MO, United States

**Keywords:** sialorrhea, drooling, botulinum toxin, muscular dystrophy, amyotrophic lateral sclerosis, neuromuscular

## Abstract

**Background:** Drooling related to bulbar weakness and dysfunction is a common concern in patients with neuromuscular disease. While there are numerous medications to manage sialorrhea, they are often limited by side effects and lack of efficacy. Botulinum toxin has shown to benefit ALS patients in a few studies, but there is scant data on the benefit in other neuromuscular conditions.

**Objective:** To assess the effectiveness of Botulinum toxin in reducing sialorrhea in patients with various neuromuscular disease.

**Design/Methods:** 25 patients (19M, 6F; 54.36 ± 17.09 yr) with documented neuromuscular illness and concern for drooling was followed for 6 weeks after Botulinum toxin injection. These patients had one of the following diagnoses: Duchenne muscular dystrophy (3), myotonic dystrophy (3), oculopharyngeal muscular dystrophy (1), inclusion body myositis (2), primary lateral sclerosis (1), amyotrophic lateral sclerosis (9), spinal muscular atrophy type 2 and 3 (2), spinal-bulbar muscular atrophy (2), and Becker's muscular dystrophy (2). A subjective drooling scale (1: markedly worse, 5: markedly better) and drooling thickness score (0=normal, 100=thick) was calculated on these patients prior to the injection and 4 and 6 weeks after the injection. Botulinum toxin 20–30 units were injected into bilateral parotid gland (70% of the dose) and submandibular gland (30% of the dose).

**Results:** The drooling thickness score at before the injection was 75.2 ± 10.46. At 4 and 6 weeks, average scores reduced to 47.2 ± 6.14 and 18.8 ± 5.26, respectively (*p* < 0.05). The average pre injection perception about drooling was 3.0 (*p* < 0.05). The average change in perception was +0.84 and +1.28 at 4 and 6 weeks, respectively, (*p* < 0.05) implying significant improvement. There were no reported adverse effects.

**Conclusion:** This study provides preliminary evidence for the use of botulinum toxin for refractory sialorrhea for a variety of neuromuscular conditions.

## Introduction

Drooling or sialorrhea is a debilitating symptom which occurs due to the presence of excess saliva in the mouth, leading to spillover out of the oral cavity. It can be either due to increased salivary production or the failure of mechanisms that clear saliva from the oral cavity (1). It is a common phenomenon in normally developed babies but is considered abnormal beyond the age of 4 years (2). The most common cause of pathologic sialorrhea in adults is Parkinson's Disease (PD) (3), with 70–80% of PD patients demonstrating sialorrhea (4). It is also commonly seen in neuromuscular disorders such as Amyotrophic Lateral Sclerosis (ALS) and Muscular Dystrophies. Swallowing is a complex neuromuscular activity which includes a voluntary oral phase and involuntary pharyngeal and esophageal phases (5), and drooling in these patients is a manifestation of the inability to clear normal secretions due to oropharyngeal muscle dysfunction rather than an increase in salivary flow (6). This can further lead to complications such as dehydration, malnutrition and aspiration pneumonia (6).

There are various modalities of treatment that can be used to manage sialorrhea. Conservative treatments include oral-motor exercises, intra oral palatal training devices and changes in diet (7). Oral anticholinergic agents such as glycopyrrolate and benztropine have also been used, as they decrease salivary production by down-regulating acetylcholine. However, the anticholinergic side effects of these medications limit their use in a number of elderly patients (8). Various studies have reported the efficacy of Botulinum Toxin (BTX) injections into the parotid and submandibular glands in the management of sialorrhea in adult patients with PD and neuromuscular disorders, and currently three type A and one type B toxin are FDA approved for this purpose (9–12). Surgical techniques such as salivary gland excision, denervation and ligation of salivary ducts are reserved for refractory cases (13).

In this study, we assess the efficacy of BTX-A administration in managing sialorrhea in adult patients with ALS and muscular dystrophies.

## Materials and Methods

Our study is a retrospective chart review of patients that attended a University based hospital approved by the Institutional review board (IRB). The study population included patients with sialorrhea due to different causes aged 18 years or more who were undergoing care at our hospital for the same. The patient population included patients suffering from Duchenne muscular dystrophy (DMD), myotonic dystrophy (MD), oculopharyngeal muscular dystrophy(OPMD), inclusion body myositis (IBM), primary lateral sclerosis (PLS), amyotrophic lateral sclerosis (ALS), spinal muscular atrophy type 2 and 3 (SMA 2 and 3), spinal-bulbar muscular atrophy(SBMA), and Becker's muscular dystrophy (BMD).

These patients had undergone BTX-A administration for managing sialorrhea by the same physician. They had received injections regularly to coincide with their clinic visit for follow up. A total of 25 patients fulfilled the criteria and were made a part of the study. These patients were treated with 2 or more medications for the complaint.

Information including age, gender, race, cause of sialorrhea, scoring on a subjective drooling scale (1: markedly worse, 5: markedly better) and scoring on a drooling thickness score (0=normal, 100=thick) was calculated on these patients prior to the injection and 4 and 6 weeks after the injection. Botulinum toxin 20–30 units were injected into bilateral parotid gland (70% of the dose) and submandibular gland (30% of the dose).

The analysis of the data included summarizing patient demographics and salivary thickness scale score in form of descriptive statistical variables including mean, standard deviation and ranges. Comparison of the salivary thickness scale at different intervals was done using Wilcoxon signed rank test and a correlation between them was done. All statistical analyses were done using SPSS v22 software (IBM, Armonk, NY).

## Results

The mean age of the population we studied was 54.36 ± 17.09 years with 76% men. The cause of drooling in majority of this patient group included ALS (36%), MD (12%) and DMD (12%). Other demographic and clinical information of the patient population is summarized in Table 1.

**Table 1:** A summary of demographic and clinical characteristics of patients in our study.

**Characteristics**	**Details**
Average age	54.36 ± 17.09 years
Gender (M/F)	19/6
Race (C/AA)	23/2
**Cause of sialorrhea**	
DMD	3
MD	3
OPMD	1
IBM	2
PLS	1
ALS	9
SMA 2	1
SMA 3	1
SBMA	2
BMD	2

*In the table, M, male; F, female; C, Caucasian; AA, African American; DMD, Duchenne muscular dystrophy; MD, myotonic dystrophy; OPMD, oculopharyngeal muscular dystrophy; IBM, inclusion body myositis; PLS, primary lateral sclerosis; ALS, amyotrophic lateral sclerosis; SMA 2 and 3, spinal muscular atrophy type 2 and 3; SBMA, spinal-bulbar muscular atrophy; and BMD, Becker's muscular dystrophy*.

**Figure 1:**
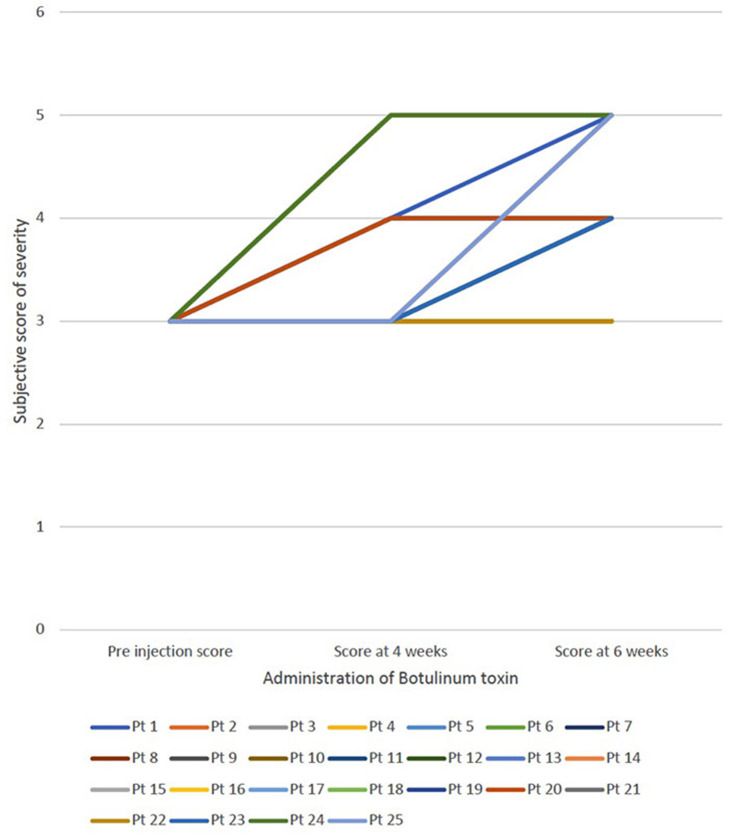
Demonstrates the change in subjective score of severity of sialorrhea at 3 different periods—preinjection, at 4 weeks and at 6 weeks for each patient.

**Figure 2:**
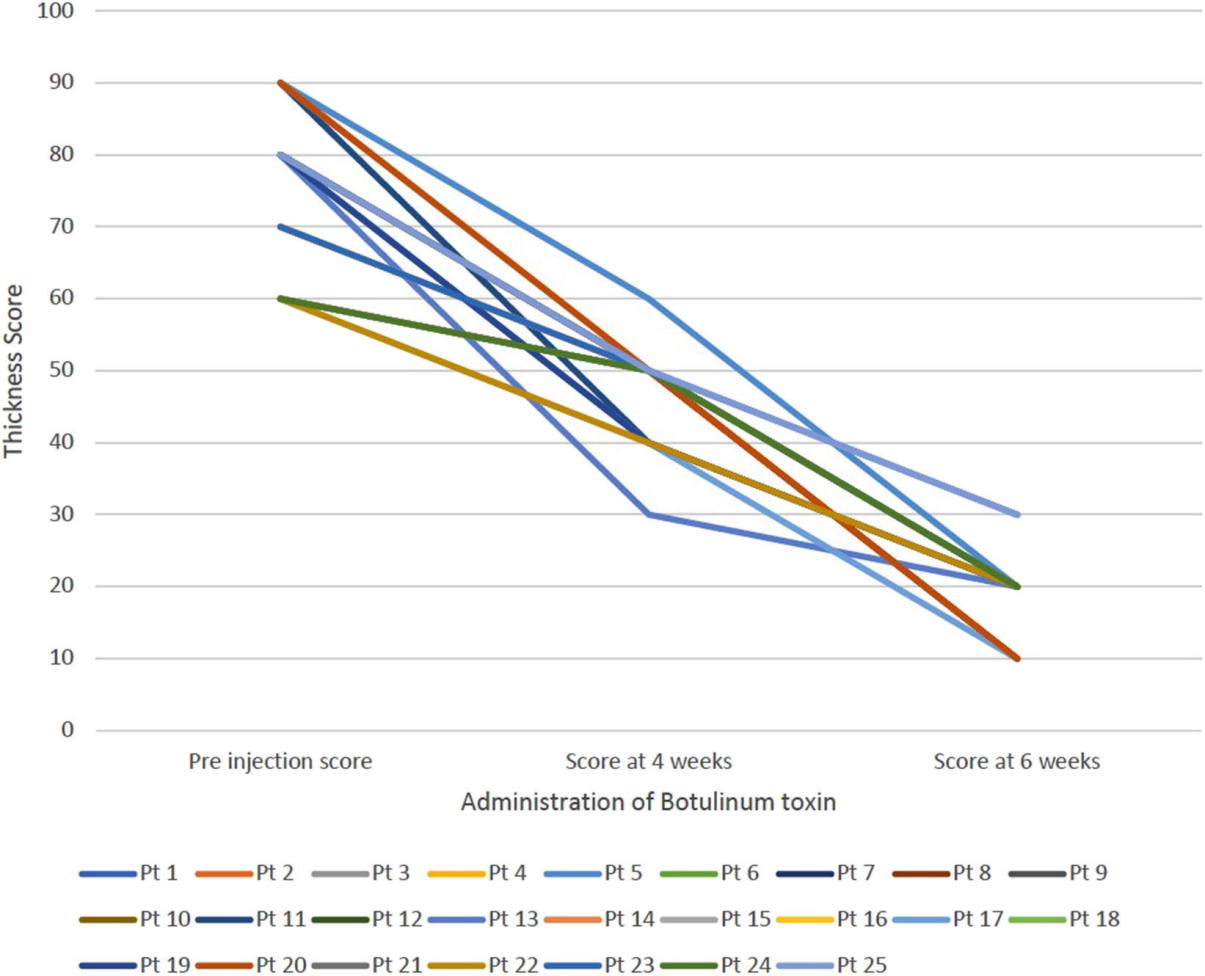
Demonstrates the change in the thickness score for each patient at three intervals—pre injection, at 4 weeks and at 6 weeks.

For all patients, botulinum toxin was well-tolerated and no adverse effects were reported. The average subjective score of severity of sialorrhea was 3, 3.84 ± 0.8 and 4.28 ± 0.6 in the pre administration of botulinum toxin, 4 weeks follow up and 6-week follow up, respectively. Figure 1 shows the change in subjective score of severity of sialorrhea at pre injection period, at 4 weeks and at 6 weeks for each patient. The average objective thickness score during these time periods were 75.2 ± 10.45, 47.2 ± 6.13 and 18.8 ± 5.25, respectively, with a *p* < 0.01. Figure 2 shows the change in objective score of thickness for each patient at preinjection period, 4 weeks and 6 weeks. The two cases where no improvement was seen in the subjective scale suffered from ALS while that where minimal change was seen in the thickness score was seen in the case of myotonic dystrophy. Patients suffering from ALS, DMD, SMA type 2, and SMA type 3 showed the most improvement on the subjective scale whereas a patient with ALS showed maximal improvement according to the thickness score.

## Discussion

While there have been multiple studies that have investigated treatment options for sialorrhea in ALS patients, there still isn't a set guideline for management (14). Our study provides preliminary evidence that BTX-A could be used as a therapeutic approach for patients of ALS and other neuromuscular diseases. As our results indicate, we found improved patient outcomes with BTX-A treatment both subjectively and objectively. 23 out of 25 patients expressed improvement in their drooling 6 weeks after the injection. Furthermore, there was a decrease in the drooling thickness score for every patient at 4 and 6 weeks. This is an important observation as drooling, among others, continues to be a disabling symptom for many patients (15).

Other treatment options for sialorrhea in neuromuscular patients include anticholinergic drugs, radiation therapy and surgical manipulation of salivary glands (15). Anticholinergic drugs are limited by side effects (16). Radiation therapy lacks consensus on the type of radiation and optimal dose (17). Lastly, while surgical procedures of the salivary duct and gland showed promise, they were not recommended due to the low life expectancy of the patients (14). Botulinum toxin B has also been studied in the management of sialorrhea, but the data has been found to be inconclusive (18).

BTX A improves sialorrhea through decreasing the release of acetylcholine at neurosecretory junctions. In addition to the efficacy, the safety and tolerability of BTX-A should be considered. The patients in our study had no adverse effects from treatment. Our data suggests a potential role of BTX-A for management of refractory sialorrhea. Limitations of the study include small sample size and disease variability. Additionally, the two scales we used for data collection are not standardized and specific to our institution. Additional studies with a randomized controlled trial containing a larger sample size and standardized scales are needed.

## Conclusion

BTX-A is an efficacious and safe option to manage sialorrhea due to a variety of neuromuscular causes.

## Data Availability Statement

The datasets generated for this study will not be made publicly available. The dataset includes deidentified patient details. Requests to access the dataset can be directed to the corresponding author.

## Ethics Statement

This retrospective chart review involving data from patients were reviewed and approved by the Institutional Review Board (IRB), University of Missouri, Columbia.

## Author Contributions

RG and HS designed the study. RG was involved in data collection. TM conducted the data analysis. HS, YN, and TM were involved in manuscript drafting.

## Conflict of Interest

The authors declare that the research was conducted in the absence of any commercial or financial relationships that could be construed as a potential conflict of interest.
